# Optimization of embryonic thermal programming confirms increased liver fattening in mule ducks and changes in lipid metabolism

**DOI:** 10.3389/fphys.2023.1142398

**Published:** 2023-05-18

**Authors:** C. Andrieux, M. Marchand, L. Larroquet, V. Veron, S. Biasutti, M. Morisson, V. Coustham, S. Panserat, M. Houssier

**Affiliations:** ^1^ Univ Pau and Pays Adour, E2S UPPA, INRAE (Institut National de Recherche pour l’Agriculture, l’Alimentation et l’Environnement), NUMEA (Nutrition Métabolisme et Aquaculture), Mont de Marsan, France; ^2^ Univ Pau and Pays Adour, E2S UPPA, IUT Génie Biologique, Mont de Marsan, France; ^3^ GenPhySE, Université de Toulouse, INRAE (Institut National de Recherche pour l’Agriculture, l’Alimentation et l’Environnement), ENVT (Ecole Nationale Vétérinaire de Toulouse), Castanet Tolosan, France

**Keywords:** liver, programming, metabolism, duck, temperature

## Abstract

**Introduction:** The embryonic thermal programming (TM) in birds has been shown to impact several physiological parameters such as resistance to thermal stress, muscle growth or immunity. In mule ducks, it has recently been shown that TM can induce metabolic programming resulting in increased liver weight and fat storage after overfeeding. However, a decrease in hatchability and *foie gras* quality was also observed, suggesting that this technique needs to be optimized. Here, we tested a new thermal manipulation condition determined with the objective of avoiding negative impacts while maintaining or improving liver properties.

**Methods:** The eggs of the control group were incubated at 37.6°C during the whole incubation period while those of the experimental group (TM group) were incubated at 39.3°C 16 h/24 h from the 11^th^ day of incubation to the 21^st^. After hatching, all the animals were fed and raised under the same conditions until the age of 12 weeks. At this stage, one part of the animals was overfed and then slaughtered 2 h (to measure rapid changes in metabolism) or 10 h after the last meal (to obtain the best technological yields), while the other part was ration-fed and slaughtered 2 h after the last meal, at the same age.

**Results:** An 8% increase in *foie gras* production was measured in the TM group compared to the control group without altering the quality of the final product (nor hatchability), confirming the successful optimization of the metabolic programming. Interestingly, these results allowed us not to reject the previously suggested hypothesis of a potential delay in metabolic processes involved in liver fattening in programmed animals, in particular by measuring a trend reversal regarding the amount of total hepatic lipids in both groups at 2 h and then 10 h after the last meal.

**Discussion:** This study therefore validates the optimization of metabolic programming by embryonic thermal manipulation for duck liver fattening. The understanding of the mechanisms of embryonic thermal programming in birds remains today very incomplete and the search for epigenetic marks (main hypothesis of the concept of programming) at the origin of the observed phenotypes could be the next step of this work.

## 1 Introduction

Heat stress is an increasingly studied factor in many livestock species as climate change threatens to increase the number of periods with large temperature variations, and thus reduce animal health and agricultural productivity (Guo et al., 2018; Alfonso et al., 2021; Wankar et al., 2021; Goel et al., 2022). Fast-growing chicken are particularly sensitive to heat stress due to the absence of sweat glands and limited development of cardiovascular and respiratory systems (Brugaletta et al., 2022). About 30 years ago, a new strategy emerged to acclimate chicken embryos to heat stress by increasing egg incubation temperature to improve postnatal thermosensitivity (Loyau et al., 2015). Since then, this method used in different avian species have shown that a change in temperature during the critical period of embryogenesis can result in various phenotypes during the later life of the animal, known as a programming indicator. In chicken, the timing, the duration and the intensity of the thermal manipulation had to be accurately chosen according to the purpose of the programming and to avoid deleterious effects (Piestun et al., 2009; Loyau et al., 2015; Al-Rukibat et al., 2016; Tainika, 2022). This concept of embryonic thermal programming has been used in broiler field to improve their thermoregulation and resistance to heat shock during the rearing period (Yahav and McMurtry, 2001; Loyau et al., 2015; Tainika, 2022). It was also shown that this type of programming could improve other performances such as meat production in broiler (Piestun et al., 2011) or more recently *foie gras* production in mule duck. Indeed it was demonstrated that three different conditions of increasing the incubation temperature, between 1°C and 1.5°C during 14 embryonic days, allowed to enhance the production of *foie gras* up to 16% (Massimino et al., 2019). In various animal species, carbohydrate and lipid metabolisms are directly impacted by heat stress (Baumgard and Rhoads, 2013), and these results suggested that liver energy metabolism may also be programmed by embryonic thermal manipulation in ducks. Nevertheless, this duck liver fattening study also showed that the two most intense conditions reduced the hatchability rate as well as the quality (melting rate) of the final product. Therefore, we conducted two studies to identify the best conditions for embryonic thermal manipulation in order to optimize liver fattening and energy metabolism programming. In the first one, we described the ontogeny of hepatic energy metabolism during mule duck embryogenesis, revealing several potentially interesting windows for a thermal stimulus (Massimino et al., 2020). In the second study, five embryonic programming windows were tested, revealing a greater fragility of duck embryos to temperature increase during the earliest stages (Massimino et al., 2019; Andrieux et al., 2022). Based on these results, we determined a ten embryonic day period with a discontinuous (16 h/24 h) increase in temperature to design our new thermal programming condition in mule ducks. We therefore applied here an increase of 1.7°C from the 11th day (E11) to the 21st day (E21) of incubation, more intense but shorter than in our previous studies, in order to optimize metabolic programming and liver fattening previously observed.

In chicken, the improved thermoregulation induced by embryonic thermal manipulation may be partly explained by a decrease in energy metabolism in the muscle, as measured by differential gene expression during heat stress (Loyau et al., 2016). Concerning duck liver fattening, a recent study (Massimino et al., 2021a) also showed that six target genes were differentially expressed around the overfeeding period in the livers of TM animals compared to controls, which may at least partly explain the greater fattening. However, this low number of molecular targets led us to hypothesize that the postprandial timing of tissue sampling (10 h after the last meal) may not have been the most appropriate. Indeed, two studies that measured kinetic molecular responses of mule duck liver up to 12 h after the last overfeeding meal, demonstrated that most of the gene responses took place in the first 5 h after the meal (Tavernier et al., 2017a; Tavernier et al., 2017b). According to these studies, we determined that gene expression analyses in particular could be more informative 2 h after the last meal.

Given all this information, we applied in this study a thermal programming of +1.7°C, 16 h/24 h from E11 to E21, and maintained liver sampling 10 h after the last meal to compare overall performances to previous studies, while adding sampling 2 h after the last meal to increase our chances to better understand the molecular mechanisms through the identification of genes altered in the short term.

## 2 Materials and methods

### 2.1 Animals

A total of 1,500 mule duck eggs (750 eggs per group) of the H85 genotype were provided by Grimaud Frères Selection company (Roussay, France), with a total average incubation period of 30 days.

**FIGURE 1:**
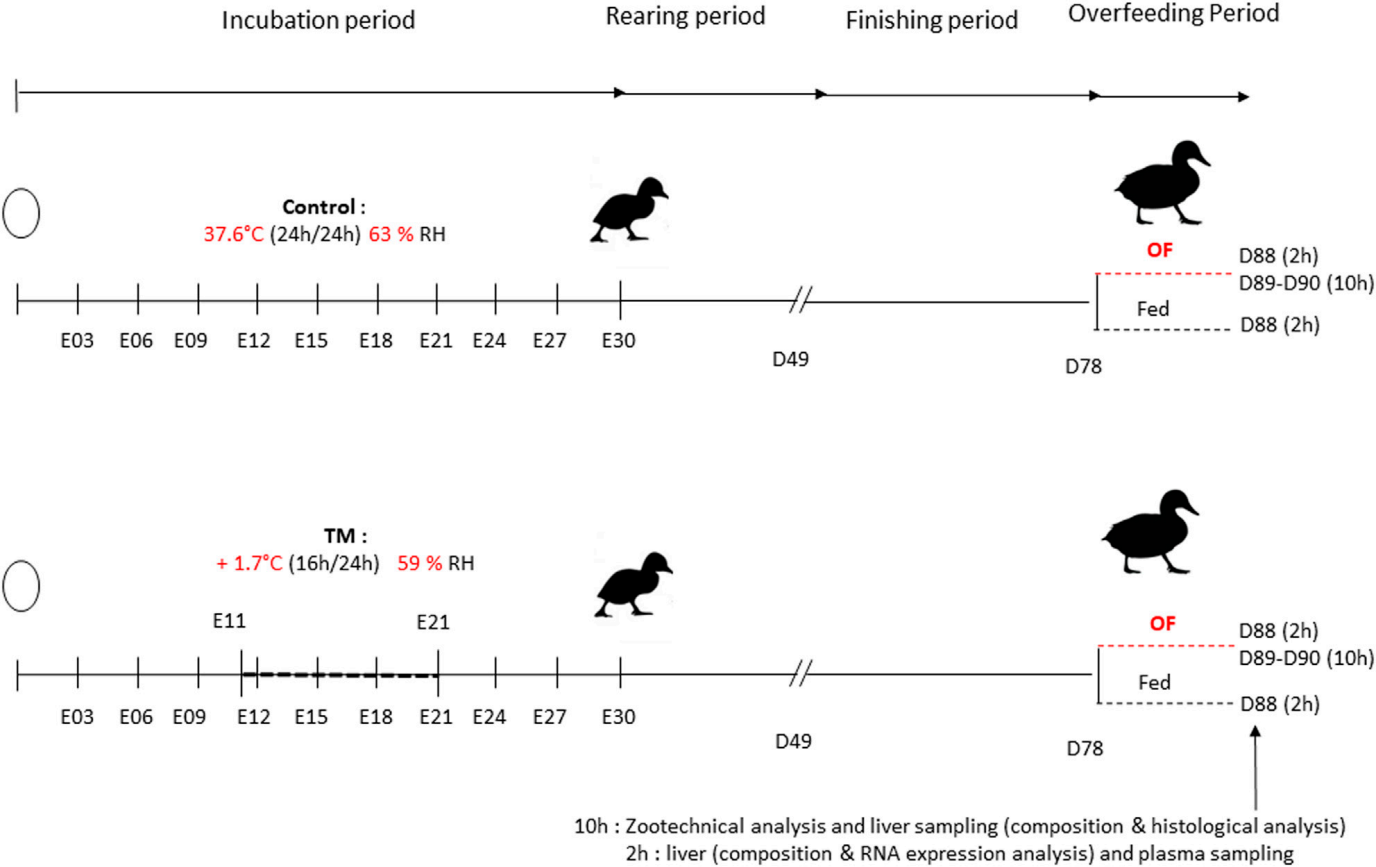
Global experimental design. The chronology of the two treatments was schematized on this time line representing the incubation time of the eggs, the rearing period and the overfeeding period of mule duck. Changes in conditions (+1.7°C 16 h/24 E11-E21 58.6% RH) applied for 10 days was illustrated by the horizontal dotted line representing a 10-day interval. A control group was present with a temperature of 37.6°C and a mean relative humidity (RH) of 63.2%. The finishing period started at 49 days (7 weeks) and the overfeeding period started at 78 days (D78) until the slaughtering day (D88, D89 or D90) where plasma and liver samples were collected. A subdivision of group was done at D78, with animal overfed and animal ration-fed as during the finishing period. Samples 10 h after the last meal were taken to measure overall performance, liver composition and histological analysis and samples 2 h after the last meal were taken for genetic relative expression, plasma analysis and liver composition.

Incubation first took place inside two identical incubators with regulation of temperature, humidity and ventilation (SOLOGNE model of LA NATIONALE reconditioned model). During the whole incubation, temperature and relative humidity (RH) were recorded every 15 min by two independent sensors (LoRa^®^ SPY U). Each incubator had its own pair of sensors. All eggs were subjected to an automatic rotation of 90° every 3 hours and were sprayed manually the afternoon before the incubation conditions were changed. In the first incubator, the temperature was maintained at 37.6°C and RH around 63.2% throughout the entire incubation period, representing the control group. In the second incubator, an embryonic thermal manipulation (TM) was done with a temperature increase of +1.7°C (39.3°C), 16 h/24 from the 11th to the 21st embryonic day (E11-E21) as well as a RH around 58.6% (Figure 1).

One light-check, called candling, was done during the incubation to estimate the mid-embryonic mortality just before the transfer to hatchery (E28). Dead eggs were removed from incubators and the remaining eggs were pooled to avoid local temperature disturbance. All eggs were moved into the same hatcher (BRETAGNE model of LA NATIONALE, reconditioned model) maintained at 37.3°C and 80% of RH during 4 days. Once a day, the number of newly hatched eggs was recorded. Hatchability (%) was calculated by the ratio of the number of ducklings born in total on the 4 days to the number of eggs put inside incubator the first day, multiplied by 100.

A total of 120 male ducklings for each group were reared under the same life conditions and fed *ad libitum* with a starting diet until 4 weeks of age. Ducks were rearing in the same house but in eight separate pens to have a density of 0.6 m^2^/duck (30 ducks in each pen of 18 m^2^). Then, they were fed with a growing diet *ad libitum* from 4 to 7 weeks of age and hourly rationed from 8 to 12 weeks, with the same diet. At 12 weeks, each treatment was divided into two groups based on similar weight (56 ducks in control group and 86 in TM group), (Figure 1). One group was subjected to overfeeding with a powder diet (46 ducks in control group and 71 in TM group) and the other group was still hourly rationed (ration-fed) with the growing diet (10 ducks in control group and 15 in TM group). The overfeeding period was characterized by a duration of 11 days, corresponding to two forced meals (by gavage) per day for a total of 21 meals (53% corn and 47% water, Palma Maisadour). The first forced meal started with 225 g per animal and ended with an amount of 460 g per animal at meal 21.

The slaughter at day 88 (D88) was done 2 h after the last meal for overfed ducks (10 ducks in control group and 13 in TM group) and for ration-fed ducks (10 ducks in control group and 15 in TM group). Slaughters at D89 and D90 days for overfed ducks were carried out 10 h after the last meal (36 ducks in control group and 58 in TM group). Ducks were killed by bleeding after stunning in an electric water bath in line with European Council Regulation (Ec) No 1099/2009 (2009) (Council Regulation (EC) No 1099/2009 of24 September 2009 on the protection of animals at the time of killing Text with EEA relevance) at the Experimental Station for Waterfowl breeding (INRAE, Artiguères, France).

### 2.2 Sampling and measurements

Mid-embryonic mortality was recorded during incubation (E28) and late embryonic mortality, sex ratio, hatchability and body weight were measured at hatch. Body weights were also recorded at the slaughtering day (D88 or D89 or D90).

At D88 (2 h after the last meal) and at D89 and D90 (10 h after the last meal), pieces of liver were sampled in the middle of the large lobe and stored at −80°C. Gene relative expressions (10 samples in control overfed and ration-fed groups, 15 in TM ration-fed group and 13 in TM overfed group) only concern samples from 2 h after the last meal. Liver lipid content and glycogen content measurements were done for all groups (9 samples in each group for lipids content, 6 samples in control ration-fed and overfed groups and 9 samples in TM groups for glycogen content). Finally, liver samples from 10 h after the last meal were immediately fixed in 4% paraformaldehyde for histological analyses (5 samples in each group). At D89 and D90 (10 h after the last meal), liver, breast muscle, leg muscle, abdominal fat and subcutaneous adipose tissue (SAT) were weighed for all the animals (36 ducks in control overfed group and 58 in TM overfed group).

Moreover, blood samples were collected from ducks slaughtered 2 h and 10 h after the last meal in disodium EDTA tubes, after carotid section.

### 2.3 Histological analysis

Liver (10 h after the last meal, when the liver weight is heaviest) were fixed in formaldehyde (4%) during 24 h, dehydrated by successive ethanol baths, with a croissant concentration, before xylene baths and inclusion in paraffine. Liver cut thickness varied between 7 and 10 µm and were performed on KNITELL lames. Slide coloration were performed by the 2-coloration method combination: PAS (Periodic Acid Shiff) and HE (Hematoxyline Eosin).

 Measurements of number of nuclei per µm^2^ and lipid droplet surface (µm^2^) were performed on 5 livers from different individuals for each group. For each liver, two slides and 4 areas per slide were used to perform the measurements (corresponding to 80,000 μm^2^ counted for each animal). The average number of nuclei per unit area was considered for each slide (n = 10 slides for each group), and the area of each identifiable lipid droplet over a total area of 80,000 μm^2^ was measured for each animal. Lipid droplets size were divided into four categories,< 100 µm^2^, between 100 and 320 μm^2^, between 320 and 640 μm^2^ and >640 μm^2^ according to (Théron et al., 2011) and their average surface area were statistically compared by the Wilcoxon test.

### 2.4 Plasma assays

Individual plasmas were collected for all the groups to evaluate the rapid response. They were separated by centrifugation at 2,000 g for 10 min at 4°C and frozen at −20°C for further analysis. Glycemia, triglyceridemia, cholesterol and non-esterified fatty acids (NEFA) were quantified by the colorimetric method using an enzymatic kit (Glucose GOD-POD, SOBIODA; Triglycerides GPO POD, SOBIODA; Cholesterol LD M, SOBIODA, NEFA HR2, Wako Richmond, United States).

### 2.5 Liver lipid and glycogen contents and melting rate

For nine fatty livers collected from overfed (10 h after the last meal) and ration-fed ducks, the total lipid content was extracted from 1.0 g of liver by homogenization with an electric grinder (IKA T25 digital Ultra-Turrax^®^) in chloroform methanol 2:1 (v/v) and measured by gravimetry by following Folch method (Folch et al., 1957). Triglycerides were saponified with dichloromethane and alcoholic potassium and gas chromatography (GC) after their conversion into volatile methyl ester, to obtain fatty acids profile.

Free glucose was measured from 100 mg of liver crushed with electric grinder (6–9 samples), then glycogen was hydrolyzed by using KOH 5 mol/L at 100°C during 2 h30. Free glucose and total glucose were measured by the same method as glucose in plasma. Liver glycogen corresponding to the difference between total glucose and free glucose, was evaluated for each sample and given in mg of glucose by g of liver tissue.

The melting test was done for all overfed ducks (36–58 samples, 10 h after the last meal). Samples of liver were placed in a tin can used for the melting test. The tin cans were cooked in an autoclave at 85°C for 60 min and stored overnight at 4°C. The melted fat was removed and the remaining sample was placed on absorbent paper. The melting rate was then measured as the percentage of loss from the initial liver weight.

### 2.6 Total RNA extraction, reverse-transcription, fluidigm and gene relative expression

Total RNA was isolated from 50 mg of frozen tissue according to the TRIZol protocol (Invitrogen/Life technologies). Their concentration was measured by spectrophotometry (optical density at 260 nm) using a Biotek EPOCH 2 microplate reader (Take 3 Plate), and all the samples were diluted at 100 ng/μL. The RNA reverse-transcription (RT) was done in duplicate, from 1 µg of RNA with 0.5 µL of transcriptase inverse (Super Script III Thermofisher 200 U/µL), 1 µL of random primers (Promega 500 μg/mL), 1 µL of PCR nucleotides mix (Promega 10 mM) and 1 µL RNase out (Thermofisher), The RNA reverse-transcription was performed in the CFX384 PCR machine (BioRad, United States) using the following program: 25°C/5 min, 55°C/60 min, 70°C/15 min.

Gene expression levels were determined by high throughput real-time quantitative PCR from Fluidigm (Gentyane platform, Clermont-Ferrand, France). A total of 60 genes involved in the different pathways of interest were first selected mainly on the basis of a previous study concerning the impact of thermal programming on their expression after OF (Massimino et al., 2021b). Two 96 well plates were prepared, one with samples at 5 ng/μL and the second with gene primers (46 genes analyzed) at 20 μM, listed in Supplementary Tables S1, S2. Genes were validated when their efficiency ranged from 1.85 to 2.15 calculated from cascade dilution of a pool of cDNA and after amplicon sequencing. The sample amplifications were realized in two steps by using the Fluidigm method. A first pre-amplification with Preamp Master Mix (Fluidigm) was done to normalize all samples following program: 95°C/10 min and 14 cycles of 95°C/15 s and 60°C/4 min. Pre-amplified samples were treated by exonuclease (NEB) before a TE low EDTA dilution and before moving samples to a 96 × 96 chip for Fluidigm Gene Expression Array. The reaction was made using 20× EvaGreen (Interchim) dye following the program:70°C/2,400 s, 60°C/30 s, 30 cycles of 96°C/5 s and 60°C/20 s. Real time quantitative PCR results were analyzed using the Fluidigm real-time PCR analysis software v.4.1.3.

The cDNA quantification was normalized with four reference genes (*ActB*, *EIF3*, *STAB1* and *USP9X*) selected according to the function GeNorm from the package CtrlGene of Rstudio (v.3.6.2). Moreover, the cDNA quantification was calculated according to the expression 2^−ΔΔCT^ (Livak and Schmittgen, 2001) with ∆Ct = Ct target gene—Ct reference genes (4 previous genes) and ∆∆Ct = ∆Ct sample - ∆Ct standard. The standard that determined the ∆Ct standard value corresponded to the average of Ct target gene across all groups. The 28 genes presented here are those for which the high throughput PCR amplification has allowed interpretation and which were relevant to this study (involved in energy metabolism).

### 2.7 Statistical analysis

To compare results of the two treatments, statistical analyses were carried out using R and Rstudio (version 3.6.2). Hatchability, mortalities and sex ratio were compared by using a Chi-squared test. Student tests were done, when normality and homoscedasticity were respected, for weights (body and organs), somatic-hepatic index (SHI) and other parameters at D89 and D90. For histological data (total lipid, nuclei number and lipid droplet surfaces) normality and homoscedasticity were not respected so Wilcoxon tests were applied.

Two-ways ANOVA tests were applied to Fluidigm results, plasma parameters or liver composition for all groups. Moreover, if the conditions for applying parametric test were not respected, nonparametric tests (Wilcoxon test) were applied: for free fatty acid in plasma, liver composition (total lipid, C14:0, C15:0, C18:0, PUFA n6, C22:4 n6, C22:5 n6) and gene relative expressions (FAS, GLUT2, HK2).

The data are presented as the average ± standard deviation (SD). In every case, differences between the groups were considered statistically significant if the *p*-value was below 0.05.

## 3 Results

### 3.1 Pre and post-hatching data

First, we studied the direct impact of thermal manipulation on embryonic mortality and hatching rate (Table 1). At E28, i.e., after the thermal manipulation, the mid embryonic mortality of the TM group was significantly higher than the control group (15.2% against 10.0%). In the hatcher, late embryonic mortality was equal between the two groups, as was the final hatching rate. At hatching, the sex ratio was not different between the two groups.

**TABLE 1:** Incubation and hatching measurements.

	Mid mortality (%)	Late mortality (%)	Hatchability (%)	Male proportion (%)
Control	10.0^b^	3.2	78.8	55.7
TM	15.2^a^	3.1	76.3	57.0
Animal number (in Control and TM groups)	501 and 533	451 and 454	668 and 654	433 and 435

During the incubation, the mid mortality was measured at the second candling (E28). In the hatchery, late mortality, hatchability and sex ratio were calculated. Chi^2^ tests were performed between the two groups for the different variables.

Within a column, means with no common superscript differed at *p* < 0.05.

TM: thermal manipulated.

### 3.2 Overall performances 10 h after the last meal

The performances were measured 10 h after the last meal for control and TM overfed animals. The weight of different tissues and the melting rate of the fatty livers were listed in Table 2. Only the liver weight was significantly different with an average gain of about 50 g for the TM group compared to the control group, confirmed by an increase in the hepato-somatic index (HSI). However, the melting rate of fatty livers after cooking did not show any significant difference between the two groups, remaining around 20%.

**TABLE 2:** Overall performances 10 h after the last overfeeding meal.

	Control	TM
Body weight (g)	5917 ± 357	5842 ± 322
Abdominal fat weight (g)	159 ± 32	163 ± 28
Pectoral muscle weight (g)	306 ± 27	305 ± 25
Skin weight (g)	151 ± 26	145 ± 27
Thigh muscle weight (g)	453 ± 49	437 ± 37
Liver weight (g)	601b ± 100	653a ±111
HSI (%)	10.2b ± 1.7	11.2a ±1.8
Melting rate (%)	19.0 ± 12.3	21.9 ± 12.3

10 h after the last force-feeding meal, the weight of total body, abdominal fat, pectoral and thigh muscles, skin and liver were measured for both groups. The hepato-somatic index (HSI) was calculated from liver weight and body weight (%).

Student tests were applied when the normality and the homoscedasticity were respected, otherwise Wilcoxon tests were applied. Within a row, means with no common superscript differed at *p* < 0.05.

n = 36 in control group and 58 in TM group.

**FIGURE 2:**
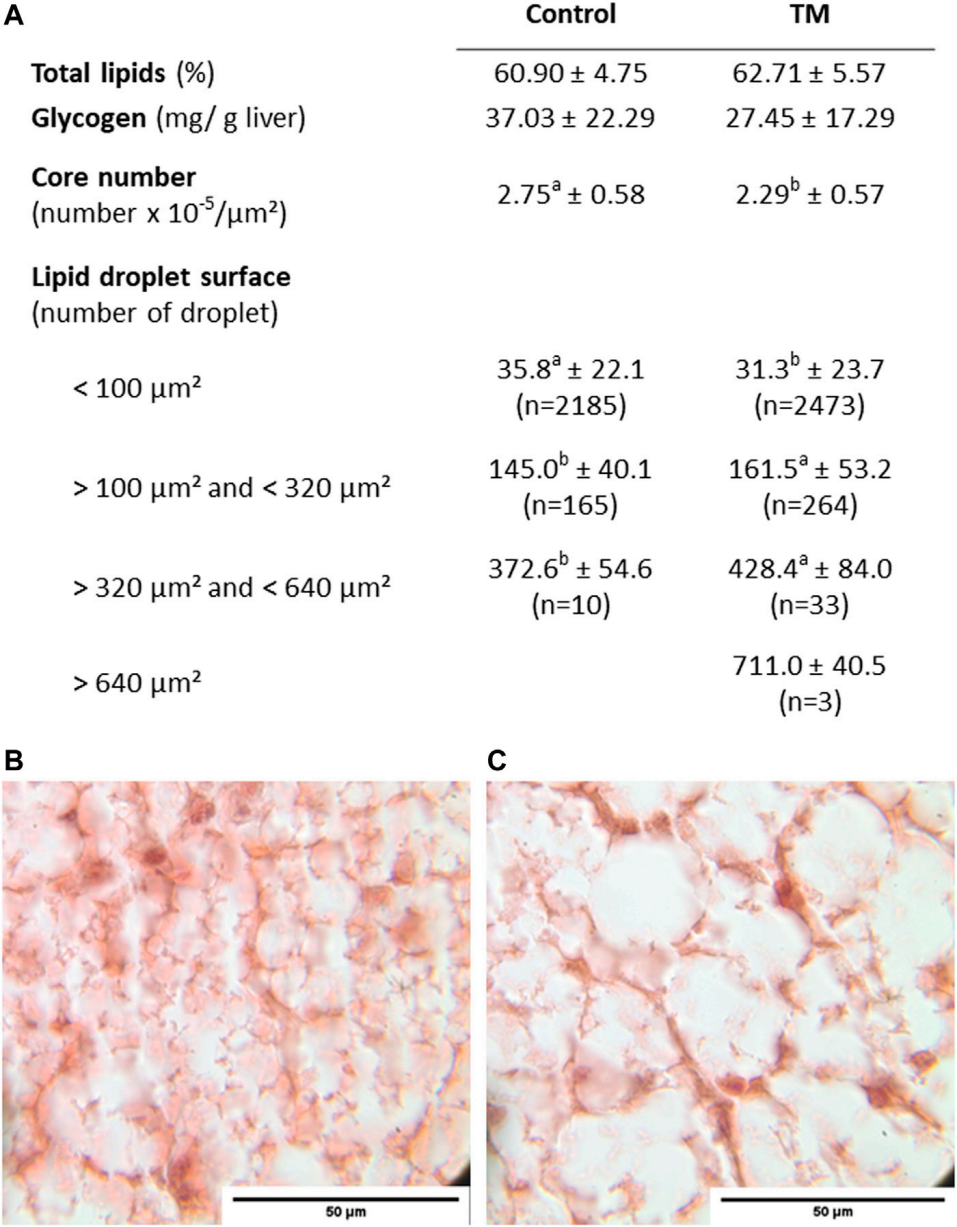
Energy composition and histological characteristics of the liver 10 h after the last overfeeding meal **(A)** Hepatic energy composition is represented by total lipid levels (%) and glycogen content (mg/g liver) corresponding to the difference between total glucose and free glucose. The histological structure is described by the number of nuclei per unit area and the number of lipid droplets classified by size into 4 subgroups (<100 μm^2^, between 100 and 320 μm^2^, between 320 and 640 μm^2^ and >640 μm^2^). Student tests were applied when the normality and the homoscedasticity were met (total lipids), otherwise (nuclei number, lipid droplet surfaces), wilcoxon tests were applied. **(B)** Histological slide of liver 10 h after the last overfeeding meal in the control group and **(C)** in the TM group. Within a row, means with no common superscript differed at *p* < 0.05. na: non applicable, ns: non significant. Liver lipid content n = 9 in each group Glycogen content n = 6 in control group and 9 in TM group.

At this sampling time, a focus on the liver energy composition revealed no significant differences between the control and TM groups in the proportion of total lipids or the proportion of glycogen (approximately 60% and 3% of liver weight respectively) (Figure 2A). Nevertheless, histological analysis of liver sections showed a significant decrease in the number of nuclei per µm^2^ in the TM group compared to the control group (2.3 × 10^−5^ vs. 2.75 × 10^−5^), confirmed by a significant decrease in the number of small-diameter droplets (<100 μm^2^) and an increase in the number of large-diameter droplets (between 100 μm^2^ and 640 μm^2^) (Figures 2A–C).

### 3.3 Plasma parameters 2 h after the last meal

Plasma levels of glucose, triglycerides, cholesterol and free fatty acids, measured 2 h after the last meal of overfeeding or daily ration were listed in Table 3. Overfeeding resulted in a significant increase in all of these parameters compared to the ration-fed ducks, with glucose levels increasing from 3.7 to 4.6 g/L for control and from 3.8 to 5.9 g/L for TM group, triglycerides from 3.2 to 5.9 g/L for control and from 3.5 to 5.9 g/L for TM group, cholesterol from 2.9 to 4.5 g/L for control and from 3.3 to 4.3 g/L for TM group and free fatty acids from 0.2 to 0.5 g/L for control and from 0.2 to 0.3 g/L for TM group. In contrast, embryonic thermal programming only had an impact on plasma glucose levels, with an overall higher level in the TM group compared to the control group.

**TABLE 3:** Plasma analysis on fed and overfed ducks slaughtered 2 h after the last meal.

	Ration-fed (2 h)		Overfed (2 h)		2 ways ANOVA			Non parametric test
	Control	TM	Control	TM	OF effect	TM effect	Interaction	
Glucose (g/L)	3.7 ± 1.0	3.8 ± 0.9	4.6 ± 0.7	5.9 ± 1.1	<0.001	0.033	ns	-
Triglycerides (g/L)	3.2 ± 1.2	3.5 ± 1.3	5.9 ± 1.1	5.9 ± 0.8	<0.001	ns	ns	-
Cholesterol (g/L)	2.9 ± 1.1	3.3 ± 0.8	4.5 ± 0.7	4.3 ± 0.8	<0.001	ns	ns	-
FFA (g/L)	0.2^b^ ± 0.1	0.2^b^ ± 0.0	0.5^a^ ± 0.3	0.3^a^ ± 0.1	-	-	-	significant

Average plasma concentrations of glucose, triglycerides, cholesterol and free fatty acid (FFA) measured 2 h after the last meal in overfed animals and animals ration-fed a daily ration (n = 9 in each group). Two-way ANOVA was applied when normality, non-correlation and homoscedasticity of residuals were met. Otherwise (FFA) a Wilcoxon test was applied to compare all conditions, with differences represented by different superscript letters in the table.

TM: thermal manipulated, ns: non significant.

### 3.4 Hepatic metabolic response 2 h after the last meal

To study the impact of embryonic thermal programming at the molecular level during overfeeding, we measured the expression of over 40 genes in the liver of overfed animals (OF) and animals fed a daily ration (Ration-fed), 2 h after their last meal. Gene expression results are listed and classified by metabolic pathway in Tables 4, 5.

**TABLE 4:** Relative expressions of genes involved in lipid and carbohydrate metabolism in the liver of ration-fed and overfed ducks slaughtered 2 h after their last meal (2 h).

	Ration-Fed (2 h)		Overfed (2 h)		2 ways ANOVA			Nonparametric test
	Control	TM	Control	TM	OF effect	TM effect	Interaction	
Lipid synthesis								
FAS	1.06 ± 0.45	1.15 ± 0.46	1.12 ± 0.41	1.00 ± 0.44	-	-	-	ns
DGAT2	0.74 ± 0.63	0.28 ± 0.18	3.06 ± 1.69	3.68 ± 2.08	<0.001	ns	ns	-
ACC	0.68 ± 0.11	1.10 ± 0.27	1.04 ± 0.35	1.29 ± 0.40	0.03	0.01	ns	-
SCD1	0.78 ± 0.30	0.84 ± 0.32	1.35 ± 0.68	1.26 ± 0.27	0.004	ns	ns	-
ACLY	0.82 ± 0.40	0.63 ± 0.37	1.49 ± 0.40	1.50 ± 0.70	0.0002	ns	ns	-
ELOVL6	0.60 ± 0.24	0.83 ± 0.30	1.56 ± 0.57	1.52 ± 0.51	<0.001	ns	ns	-
PLIN2	0.34 ± 0.13	0.55 ± 0.30	2.87 ± 0.84	2.21 ± 0.63	<0.001	ns	ns (0.05)	-
CEPT1	0.73 ± 0.41	1.80 ± 0.41	0.95 ± 0.33	1.34 ± 0.79	ns	0.002	ns	-
ACAT1	1.24 ± 0.80	1.82 ± 1.05	0.56 ± 0.26	1.06 ± 0.45	0.006	0.006	ns	-
ME	0.87 ± 0.92	0.65 ± 0.35	1.66 ± 0.57	2.12 ± 0.89	<0.001	ns	ns	-
GPAT1	0.74 ± 0.44	0.92 ± 0.66	1.41 ± 0.66	1.39 ± 0.50	0.01	ns	ns	-
**Lipid oxidation**								
ACOX1	0.75 ± 0.31	1.12 ± 0.22	1.12 ± 0.36	1.08 ± 0.39	ns	ns	ns	-
ACAD11	0.90 ± 0.71	3.29 ± 2.63	0.33 ± 0.19	2.93 ± 3.39	ns	0.002	ns	-
ACSL1	1.17 ± 0.55	1.04 ± 0.64	0.88 ± 0.39	0.92 ± 0.39	ns	ns	ns	-
LIPC	0.98 ± 0.68	2.74 ± 1.09	0.56 ± 0.29	0.83 ± 0.48	0.003	0.004	ns	-
ALDH7A1	0.82 ± 0.34	1.29 ± 0.21	1.10 ± 0.27	0.97 ± 0.41	ns	ns	0.02	-
**Lipid transport**								
APOB	1.18 ± 0.28	1.64 ± 0.29	0.72 ± 0.25	0.85 ± 0.10	<0.001	0.002	ns	-
**Regulator**								
ChREBP	1.15 ± 0.76	1.05 ± 0.53	0.92 ± 0.20	1.26 ± 0.54	ns	ns	ns	-
LXRA	1.10 ± 0.63	1.67 ± 0.66	0.85 ± 0.32	0.98 ± 0.43	ns	0.03	ns	-
PPARA	1.19 ± 0.45	1.70 ± 0.52	0.85 ± 0.31	0.75 ± 0.28	<0.001	ns	ns	-
PPARg	1.14 ± 1.06	0.51 ± 0.26	2.07 ± 1.33	1.20 ± 0.69	0.008	0.01	ns	
**Carbohydrate oxidation**								
PDHA1	0.87 ± 0.39	1.23 ± 0.58	1.26 ± 0.43	0.95 ± 0.23	ns	ns	0.03	-
GAPDH	0.77 ± 0.23	1.03 ± 0.30	1.14 ± 0.36	1.36 ± 0.61	0.02	ns	ns	-
HK2	1.40 ± 0.28	0.82 ± 0.61	1.04 ± 0.19	1.47 ± 1.18	-	-	-	ns
ENO1	0.70 ± 0.28	1.15 ± 0.42	1.12 ± 0.26	1.33 ± 0.53	0.04	0.03	ns	-
HK1	1.25 ± 0.68	0.68 ± 0.42	1.59 ± 0.54	1.04 ± 0.45	ns	0.009	ns	-
CREB2 ATF4	1.08 ± 0.56	1.57 ± 0.45	0.98 ± 0.44	0.88 ± 0.45	0.03	ns	ns	-
**Carbohydrate transport**								
GLUT2	1.13 ± 0.33	0.90 ± 0.22	1.21 ± 0.62	1.23 ± 0.86	-	-	-	ns

Average of relative expressions of genes classified by metabolic pathway in ration-fed and overfed animals (n = 6–8).

Two-way ANOVA was applied when normality, non-correlation and homoscedasticity of residuals were respected. Otherwise (*FAS, GLUT2* and *HK2*), student and wilcoxon tests were applied to compare all conditions and significant differences were represented by superscript letters in the table.

TM: thermal manipulation; OF: overfeeding; ns: non significant.

**TABLE 5:** Overall energy composition and detailed lipid content of the liver of ration-fed and overfed ducks slaughtered 2 h after the last meals (2 h).

	Ration-fed (2 h)		Overfed (2 h)		Statistics	
	Control	TM	Control	TM	2 way ANOVA	Non parametric test
**Glycogen** (mg/g of liver)	75.7 ± 33.1	69.9 ± 25.8	30.2 ± 8.7	45.1 ± 12.8	OF effect: *** TM effect: ns Interaction: ns	-
**Total lipids** (%)	6.6^c^ ± 1.0	6.3^c^ ± 0.6	58.0^a^ ± 3.7	51.3^b^ ± 6.6	-	significant
**Saturated FA** (%)	46.6 ± 2.7	44.5 ± 2.5	42.7 ± 2.3	40.2 ± 1.8	OF effect: *** TM effect: ** Interaction: ns	-
C14:0	0.6^b^ ± 0.1	0.5^b^ ± 0.1	0.8^a^ ± 0.1	0.9^a^ ± 0.2	-	significant
C15:0	1.5 ± 1.3	1.6 ± 1.4	nd	nd	-	ns
C16:0	28.7 ± 2.7	27.1 ± 2.3	25.7 ± 2.0	25.6 ± 1.3	OF effect: *** TM effect: ns Interaction: ns	-
C18:0	15.7^ab^ ±1.9	15.2^ab^ ± 1.8	16.0^a^ ± 1.0	13.6^b^ ± 2.7	-	significant
C20:0	nd	nd	0.1 ± 0.0	0.1 ± 0.0	-	ns
**MUFA** (%)	26.9 ± 5.6	27.7 ± 5.2	55.6 ± 2.1	57.0 ± 1.2	OF effect: *** TM effect: ns Interaction: ns	-
C16:1	1.9 ± 0.6	2.1 ± 0.7	2.8 ±0.2	3.6 ± 1.8	OF effect: *** TM effect: ns Interaction: ns	-
C18:1	24.6 ± 5.1	25.3 ± 4.6	52.1 ± 2.1	52.9 ± 1.6	OF effect: *** TM effect: ns Interaction: ns	-
C20:1	0.3 ± 0.1	0.3 ± 0.0	0.5 ± 0.1	0.5 ± 0.1	OF effect: *** TM effect: ns Interaction: ns	-
**PUFA n3** (%)	1.8 ± 0.4	1.9 ± 0.5	nd	nd	-	ns
C16:4 n3	0.6 ± 0.2	0.7 ± 0.4	nd	nd	-	ns
C18:3 n3	0.1 ± 0.0	0.1 ± 0.0	nd	nd	-	ns
C22:5 n3	0.2 ± 0.1	0.2 ± 0.1	nd	nd	-	ns
C22:6 n3	0.8 ± 0.2	0.9 ± 0.2	nd	nd	-	ns
**PUFA n6** (%)	23.7^a^ ± 4.7	24.8^a^ ± 5.4	1.4^c^ ± 0.2	1.9^b^ ± 0.3	-	significant
PUFA 14	0.2 ± 0.0	0.2 ± 0.1	nd	nd	-	ns
C18:2 n6	7.7 ± 1.3	8.0 ± 1.7	0.9 ± 0.1	1.3 ± 0.3	OF effect: *** TM effect: ns Interaction: ns	-
C18:3 n6	0.16 ± 0.0	0.1 ± 0.0	nd	nd	-	ns
C20:2 n6	0.2 ± 0.1	0.2 ± 0.1	0.1 ± 0.0	0.2 ± 0.0	ns	-
C20:3 n6	0.8 ± 0.1	0.8 ± 0.2	0.1 ± 0.0	0.1 ± 0.0	OF effect: *** TM effect: ns Interaction: ns	-
C20:4 n6	12.2 ± 2.8	12.6 ± 3.1	0.2 ± 0.1	0.3 ± 0.1	OF effect: *** TM effect: ns Interaction: ns	-
C22:4 n6	1.1 ± 0.3	1.2 ± 0.3	nd	nd	-	ns
C22:5 n6	1.4 ± 0.4	1.6 ± 0.4	nd	nd	-	ns

Glycogen was indirectly (difference between total glucose and free glucose) measured from liver of ducks slaughtered 2 h after the last meal (n = 6 for control group and n = 9 for TM group). Total lipids (n = 9 for each group) of the liver were also measured from liver of ducks slaughtered 2 h after the last meal. Fatty acids below 0.1% have been removed.

Two-way ANOVA was applied when normality, non-correlation and homoscedasticity of residuals were respected. Otherwise (total lipids, C14:0, C15:0, C18:0, PUFA n6, C22:4 n6 and C22:5 n6), student and wilcoxon tests were applied to compare all conditions and significant differences are represented by superscript letters in the table.

OF: overfeeding; TM: thermal manipulation; nd: non determined; ns: non significant.

Among the 11 tested genes involved in lipid synthesis (Table 4), 8 were significantly increased by overfeeding (*DGAT2*, *ACC*, *SCD1*, *ACLY*, *ELOV6*, *PLIN2*, *ME* and *GPAT1*), compared to ration-fed animals measured at the same postprandial time. These increases were associated with an upregulation of the regulator *PPARG*, but only *LIPC* involved in lipid oxidation was decreased by OF under these conditions.

Concerning carbohydrate metabolism, only two genes involved in their oxidation (*GAPDH* and *ENO1*) were significantly increased by overfeeding.

In contrast, the measurement of gene expression 2 h after the last meal allowed us to measure many modulations significantly linked to embryonic thermal manipulation (TM), whether for carbohydrate or lipid metabolism. Indeed, TM significantly modulated the expression of two major regulators, the *LXRA* and *PPARg* genes, genes involved in lipid synthesis (*ACC*, *ELOVL6*, *CEPT1*, and *ACAT1 ME*), carbohydrate oxidation (*GAPDH*, *ENO1* and *HK1*, *CREB2*), but also in lipid oxidation (*ACOX1*, *ACAD11* and *LIPC and ALDH7A1*). Finally, *APOB* involved in lipid transport was also increased by TM, compared to ducks undergoing conventional incubation.

### 3.5 Lipid composition of the liver 2 h after the last meal

To assess whether these TM-induced modulations of gene expression could be related to a rapid change in liver energy composition, we then analyzed the glycogen and lipid content in the liver of ration-fed and overfed ducks 2 h after their last meal (Table 5). First, OF induced a decrease in the proportion of glycogen (from 75.7 to 30.2 mg/g of liver in control and from 69.9 to 45.1 mg/g of liver in TM group), as well as a strong increase in the proportion of total lipids (from 6.6% to 58% in control and from 6.3% to 51.3% in TM group), compared to the ration-fed animals. Interestingly, 2 h after the last meal, total lipid level in the TM group was significantly lower than in the control group (Table 5), in contrast to what was observed at 10 h after the last meal (Figure 2).

Overfeeding also induced large changes in the lipid composition of the liver. In particular, we measured a significant decrease in saturated fatty acids (SFA), mainly due to a decrease in C16:0, an increase in overall monounsaturated fatty acids (MUFA), and a large decrease in polyunsaturated fatty acids (PUFA) n6 and to a lesser extent PUFA n3. The significantly lower amount of SFA in the TM group after overfeeding compared with the control group could be explained by a significantly lower amount of C18:0. Finally, the very low amount of PUFA n3 did not allow to statistically measure the impact of the dietary challenge, but the decrease in PUFA n6 was significantly lower in the TM group than in the control group after overfeeding.

## 4 Discussion

The effects of embryonic thermal programming on the acquisition of thermotolerance are well known in poultry (Piestun et al., 2011; Piestun et al., 2013; Loyau et al., 2015), but the impact of such programming strategy on *foie gras* production in mule ducks has only recently been revealed (Massimino et al., 2019; Massimino et al., 2021a). The objectives of this study were to test a new programming condition to optimize *foie gras* production and quality and to measure a broad spectrum of molecular modulations induced by this programming. For this purpose, two types of samples were taken, the first 10 h after the last meal (to obtain optimal weight and liver quality) and the second 2 h after the last meal (to measure rapid metabolic changes).

### 4.1 The new TM improved hatching, performances and *foie gras* production

To avoid the negative impacts of embryonic thermal programming (+1.5°C 16 h/24 E11-E27) on hatching and *foie gras* quality observed in our previous study (Massimino et al., 2019), we applied a shorter thermal stimulus (10 days versus 14 days), and focused on a period of high transcriptional activity of liver metabolism (Massimino et al., 2020). This period (E11-E21) corresponds to an early developmental window but mature enough not to be too sensitive to temperature changes, and previously shown to maintain normal hatchability (Andrieux et al., 2022). The choice of increasing the temperature by 1.7°C (against 1.5°C) during this embryonic period was therefore intended to improve the metabolic programming previously described, in order to optimize the performance of the overfed mule ducks.

Thus, although we measured a significant increase in mid mortality in the TM group compared to the control, this new embryonic thermal manipulation (TM) maintained hatching performance at the same level as the control group, confirming the successful optimization of the thermal manipulation for this first criterion.

Then, regarding the performances after overfeeding, this new condition of TM has also improved the yield compared to the first study, as liver weight was significantly higher than that of the control group (about 8%), and melting rate was maintained at the same level, at around 20% therefore below the 30% threshold accepted in *foie gras* production (République française, 1993). However, here, the increase in the liver weight in the TM group was not directly related to an increase in total lipid compared with the control group, but remained associated with an increase in the number of large lipid droplets in the hepatocytes.

### 4.2 Numerous molecular modulations in liver associated with the TM

We then focused on the modulations induced by TM on gene expressions involved in hepatic metabolism. For this purpose, we chose to take samples 2 h after the last meal, in order to measure the level of activation of the pathways of interest in both groups and thus try to better understand the mechanisms of this metabolic programming. These analyses allowed us to confirm the impact OF on the hepatic metabolism of overfed ducks by comparing them to ration-fed ducks, but also to measure many effects of thermal manipulation in response to OF.

First of all, the overfeeding (OF) increased all measured plasma parameters: glucose, triglycerides, cholesterol and free fatty acid levels, as already demonstrated in different studies (Hermier et al., 2003; Marie-Etancelin et al., 2011; Tavernier et al., 2016; Tavernier et al., 2017b), compared to ration-fed animals. Animals in the TM group had significantly higher blood glucose levels than those in the control group, suggesting a change in carbohydrate metabolism such as better intestinal absorption (Marathe et al., 2015; Fiorentino et al., 2017; Trico et al., 2019), slower diffusion to consumer tissues (Dimitriadis et al., 2021) or any other change in its synthesis or oxidation/utilization. Only additional analyses on other tissues could allow to discriminate these two hypotheses.

Regarding the relative expression of genes involved in hepatic lipid metabolism, we confirmed under these conditions that OF induced an increase in the expression level of several genes involved in lipid synthesis and carbohydrate oxidation, compared to ration-fed animals but slaughtered at the same postprandial stage (2 h after their last meal). Contrary to previous studies showing a general decrease in genes involved in lipid oxidation after OF (Yahav and McMurtry, 2001; Théron et al., 2011), only *LIPC* expression was decreased in overfed animals compared with ration-fed animals in this study. These results suggest that 2 hours after a meal, whether under rationing or overfeeding conditions, lipid oxidation is globally modulated in the same way. Interestingly, TM itself significantly increased several genes involved in lipid synthesis and carbohydrate oxidation compared to the control group, supporting their greater fattening. On the contrary, TM significantly increased the expression of genes involved in lipid oxidation (*ACAD11* and *LIPC*), compared to the relative expressions of the control group, which is not consistent with greater fattening. Recently published data from our laboratory suggest that after a meal, mule ducks that have undergone TM may exhibit a temporal shift in their hepatic transcriptional response (Andrieux et al., 2023). It is therefore possible that the decrease in lipid oxidation usually induced by OF (Tavernier et al., 2016; Massimino et al., 2021b) or a rationed meal (Andrieux et al., 2023) is not yet measurable 2 h after the last meal in the control group, or simply of the same level 2 h after a force-feeding meal and 2 h after a rationed meal (compared with fasted animals). The increase in expression of genes involved in lipid oxidation measured here in the TM group compared with the control group may therefore reflect only a delay in the upcoming meal-induced decrease. It is also interesting to note that the genes involved in lipid synthesis (*ACC, CEPT1, ACAT)*, *CREB2)* or regulatory genes (*LXRA, PPARA,*) that are upregulated by TM are not exactly the same as those specifically induced by OF at that time. This observation may reinforce the idea that the response of the TM group may be phase-shifted relative to the control group.

In the previous study which investigated the impact of TM on hepatic metabolism gene expression 10 h after the last meal of only 2 genes were identified as specific targets of thermal programming (Massimino et al., 2021a). Here, we were able to confirm that sampling 2 h after the last meal allowed to identify more targets of embryonic thermal programming, as 10 genes were revealed to be specifically modulated by TM, It would therefore be very interesting and informative to study these gene expressions on postprandial kinetics in order to understand the set of regulations that may lead to increased liver fattening.

### 4.3 TM also had an impact on plasma and liver composition

In addition to changes in gene expression, samples taken 2 h after the last meal also showed alterations in the energy content of liver cells.

First of all, it is interesting to note the differences observed in glycogen and total lipid content between the samples taken 10 and 2 h after the last meal. Measurements taken 2 h after the last meal showed a significantly lower amount of lipids in the TM overfed group compared to the control overfed group, while 10 h after the last meal, the TM group had completely recovered this deficit. This decrease in total lipids measured at 2 h, is concomitant with a trend (not significant) to a greater proportion of hepatic glycogen in TM-overfed compared to control-overfed (45.1 mg/g of liver vs 30.2 mg/g of liver), while at 10 h this trend was reversed. These observations support the hypothesis of a temporal shift induced by embryonic thermal programming, which could explain a higher liver weight specifically in the TM group 10 h after the last meal.

The more precise analysis of liver lipid content, carried out only 2 h after the last meal, allowed us to highlight overfeeding effects but also embryonic thermal manipulation effects (TM). First, the proportions of SFA, MUFA and PUFA measured 2 h after the last overfeeding meal in the control group were very close to those measured even after 10 h in previous studies (Molee et al., 2005; Chartrin et al., 2006). The proportion of MUFA was strongly increased by overfeeding (from 25.6% to 56.1%) while that of PUFA was strongly decreased (from 25% to 1.5%). We recently showed that a conventional meal following a fasting period (ration-fed) could also significantly affect lipid proportions 4 h after the meal (Andrieux et al., 2023), but overall SFA, MUFA, and PUFA levels remained close to those measured in non-overfed animals (48%, 25%, and 23%, respectively). These data suggest that the high MUFA and low PUFA levels measured after OF in this present study are mainly due to the accumulation of overfeeding meals and not to the last meal effect. However, it is still possible here, 2 h after the last overfeeding meal, to measure an impact of the embryonic thermal programming, since we measured a significant decrease in the proportion of SFA in the TM group compared to the control group, partly due to a decrease of C18:0 after OF. SFAs are the first fatty acids produced during *de novo* lipogenesis (Collins et al., 2011), and then serve as substrates to produce MUFAs, which therefore accumulate during OF. Here, the decrease in SFAs measured in the TM group did not correlate with a modulation of their MUFAs, either because the cumulative effect of the OF meals was stronger than the modulation induced by TM after the last meal, or because the timing was too short (2 h after the last meal, the SFAs may not yet desaturated into MUFA). It would be very interesting in a future trial to measure lipid composition just before the last overfeeding meal, and 10 h after, to check the evolution of these modulations in hourly kinetics. This would certainly allow a better understanding of the metabolic kinetics of liver fattening in mule ducks, but also to draw conclusions about the existence of a time lag in the metabolic response in TM ducks.

## 5 Conclusion

First, we managed to optimize the embryonic thermal programming technique for *foie gras* production through new incubation conditions that allowed an increase in liver weight, while eliminating the negative effects on hatchability and final product quality previously observed. Secondly, we identified many new TM targets linked to liver composition, structure and gene relative expressions, which allowed us to open a hypothesis on the mechanism involved in the programming. These data now open up a whole field of study concerning the mechanisms at the origin of the hepatic metabolic programming, while giving tracks of research. In particular it would be interesting to perform a more thorough kinetic study to test the existence of the suggested time lag between TM and control animals while looking for epigenetic marks.

## Data Availability

The original contributions presented in the study are included in the article/Supplementary Material, further inquiries can be directed to the corresponding author.

## References

[B1] Al-Rukibat R. K. Al-Zghoul M. B. Hananeh W. M. Al-Natour M. Q. Abu-Basha E. A. (2016). Thermal manipulation during late embryogenesis: Effect on body weight and temperature, thyroid hormones, and differential white blood cell counts in broiler chickens. Poult. Sci. Assoc. Inc. 96 (1), 234–240. 10.3382/ps/pew298 27587725

[B2] Alfonso S. Gesto M. Sadoul B. (2021). Temperature increase and its effects on fish stress physiology in the context of global warming. J. Fish. Biol. 98 (6), 1496–1508. 10.1111/jfb.14599 33111333

[B3] Andrieux C. Biasutti S. Barrieu J. Morganx P. Morisson M. Coustham V. (2022). Identification of different critical embryonic periods to modify egg incubation temperature in mule ducks. Anim. [Internet] 16 (1), 100416. 10.1016/j.animal.2021.100416 34954551

[B4] Andrieux C. Marchand M. Larroquet L. Veron V. Biasutti S. Barrieu J. (2023). Fasting/refeeding: An experimental model to study the impact of early thermal manipulation on hepatic metabolism in mule ducks. Am. J. Physiol. - Regul. Integr. Comp. Physiol. 324, R45–R57. 10.1152/ajpregu.00158.2022 36315183

[B5] Baumgard L. H. Rhoads R. P. (2013). Effects of heat stress on postabsorptive metabolism and energetics. Annu. Rev. Anim. Biosci. 1, 311–337. 10.1146/annurev-animal-031412-103644 25387022

[B6] Brugaletta G. Teyssier J. R. Rochell S. J. Dridi S. Sirri F. (2022). A review of heat stress in chickens. Part I: Insights into physiology and gut health. Front. Physiol. 13, 934381–934415. 10.3389/fphys.2022.934381 35991182 PMC9386003

[B7] Chartrin P. Bernadet M. D. Guy G. Mourot J. Duclos M. J. Baéza E. (2006). The effects of genotype and overfeeding on fat level and composition of adipose and muscle tissues in ducks. Anim. Res. 55 (3), 231–244. 10.1051/animres:2006011

[B8] Collins J. M. Neville M. J. Pinnick K. E. Hodson L. Ruyter B. Van Dijk T. H. (2011). De novo lipogenesis in the differentiating human adipocyte can provide all fatty acids necessary for maturation. J. Lipid Res. 52 (9), 1683–1692. 10.1194/jlr.M012195 21677304 PMC3151688

[B9] Dimitriadis G. D. Maratou E. Kountouri A. Board M. Lambadiari V. (2021). Regulation of postabsorptive and postprandial glucose metabolism by insulin-dependent and insulin-independent mechanisms: An integrative approach. Nutrients 13 (1), 159–233. 10.3390/nu13010159 33419065 PMC7825450

[B10] Fiorentino T. V. Suraci E. Arcidiacono G. P. Cimellaro A. Mignogna C. Presta I. (2017). Duodenal sodium/glucose cotransporter 1 expression under fasting conditions is associated with postload hyperglycemia. J. Clin. Endocrinol. Metab. 102 (11), 3979–3989. 10.1210/jc.2017-00348 28938485

[B11] Folch J. Lees M. Sloane stanley G. H. (1957). A simple method for the isolation and purification of total lipides from animal tissues. J. Biol. Chem. 226 (1), 497–509. 10.1016/s0021-9258(18)64849-5 13428781

[B12] Goel A. Ncho C. M. Jeong C. M. Choi Y. H. (2022). Embryonic thermal manipulation and in ovo gamma-aminobutyric acid supplementation regulating the chick weight and stress-related genes at hatch. Front. Vet. Sci. 8, 807450. 10.3389/fvets.2021.807450 35071394 PMC8777219

[B13] Guo Z. Lv L. Liu D. Fu B. (2018). Effects of heat stress on piglet production/performance parameters. Trop. Anim. Health Prod. 50 (6), 1203–1208. 10.1007/s11250-018-1633-4 29948773

[B14] Hermier D. Guy G. Guillaumin S. Davail S. André J. M. Hoo-Paris R. (2003). Differential channelling of liver lipids in relation to susceptibility to hepatic steatosis in two species of ducks. Comp. Biochem. Physiol. - B Biochem. Mol. Biol. 135 (4), 663–675. 10.1016/s1096-4959(03)00146-5 12892758

[B15] Livak K. J. Schmittgen T. D. (2001). Analysis of relative gene expression data using real-time quantitative PCR and the 2(-Delta Delta C(T)) Method. Methods 25 (4), 402–408. 10.1006/meth.2001.1262 11846609

[B16] Loyau T. Bedrani L. Berri C. Métayer-Coustard S. Praud C. Coustham V. (2015). Cyclic variations in incubation conditions induce adaptive responses to later heat exposure in chickens: A review. Animal 9 (1), 76–85. 10.1017/S1751731114001931 25118598

[B17] Loyau T. Hennequet-Antier C. Coustham V. Berri C. Leduc M. Crochet S. (2016). Thermal manipulation of the chicken embryo triggers differential gene expression in response to a later heat challenge. BMC Genomics [Internet] 17 (1), 329. 10.1186/s12864-016-2661-y 27142519 PMC4855354

[B18] Marathe C. S. Horowitz M. Trahair L. G. Wishart J. M. Bound M. Lange K. (2015). Relationships of early and late glycemic responses with gastric emptying during an oral glucose tolerance test. J. Clin. Endocrinol. Metab. 100 (9), 3565–3571. 10.1210/JC.2015-2482 26171801

[B19] Marie-Etancelin C. Basso B. Davail S. Gontier K. Fernandez X. Vitezica Z. G. (2011). Genetic parameters of product quality and hepatic metabolism in fattened mule ducks. J. Anim. Sci. 89 (3), 669–679. 10.2527/jas.2010-3091 21075969

[B20] Massimino W. Andrieux C. Biasutti S. Davail S. Bernadet M. Pioche T. (2021). Impacts of embryonic thermal programming on the expression of genes involved in foie gras production in mule ducks. Front. Physiol. 12, 779689–779712. 10.3389/fphys.2021.779689 34925068 PMC8678469

[B21] Massimino W. Andrieux C. Biasutti S. Davail S. Bernadet M. D. Pioche T. (2021). Impact of embryonic thermal programming on the expression of genes involved in foie gras production in mule ducks. Front. Physiol. 2021. In Press. 10.3389/fphys.2021.779689 PMC8678469 34925068

[B22] Massimino W. Davail S. Bernadet M. Pioche T. Ricaud K. Gontier K. (2019). Impact of thermal manipulation during embryogenesis on hepatic metabolism in mule ducks. Fontiers Physiol. 10, 1–12. 10.3389/fphys.2019.01495 PMC6920244 31920700

[B23] Massimino W. Davail S. Secula A. Andrieux C. Bernadet M. Pioche T. (2020). Ontogeny of hepatic metabolism in mule ducks highlights different gene expression profiles between carbohydrate and lipid metabolic pathways. BMC Genomics 21, 742–813. 10.1186/s12864-020-07093-w 33109083 PMC7590481

[B24] Molee W. Bouillier-Oudot M. Auvergne A. Babilé R. (2005). Changes in lipid composition of hepatocyte plasma membrane induced by overfeeding in duck. Comp. Biochem. Physiol. - B Biochem. Mol. Biol. 141 (4), 437–444. 10.1016/j.cbpc.2005.05.007 15964231

[B25] Piestun Y. Druyan S. Brake J. Yahav S. (2013). Thermal treatments prior to and during the beginning of incubation affect phenotypic characteristics of broiler chickens posthatching. Poult. Sci. 92 (4), 882–889. 10.3382/ps.2012-02568 23472010

[B26] Piestun Y. Halevy O. Shinder D. Ruzal M. Druyan S. Yahav S. (2011). Thermal manipulations during broiler embryogenesis improves post-hatch performance under hot conditions. J. Therm. Biol. [Internet] 36 (7), 469–474. 10.1016/j.jtherbio.2011.08.003

[B27] Piestun Y. Harel M. Barak M. Yahav S. Halevy O. (2009). Thermal manipulations in late-term chick embryos have immediate and longer term effects on myoblast proliferation and skeletal muscle hypertrophy. J. Appl. Physiol. 106 (1), 233–240. 10.1152/japplphysiol.91090.2008 19023019 PMC2636946

[B28] République française (1993). Décret93-999. Décret n°93-999 du 9 août 1993 relatif aux préparations à base de foie gras. France: Journal officiel de la république française.

[B29] Tainika B. (2022). Thermal manipulation: Embryonic development, hatchability, and hatching quality of broiler chicks. intech open.

[B30] Tavernier A. Davail S. Ricaud K. Bernadet M.-D. Gontier K. (2016). Genes involved in the establishment of hepatic steatosis in Muscovy, Pekin and mule ducks. Mol. Cell Biochem. 424 (1–2), 147–161. 10.1007/s11010-016-2850-7 27796685

[B31] Tavernier A. Ricaud K. Bernadet M.-D. Davail S. Gontier K. (2017). Kinetics of expression of genes involved in glucose metabolism after the last meal in overfed mule ducks. Mol. Cell Biochem. 430 (1–2), 127–137. 10.1007/s11010-017-2960-x 28324238

[B32] Tavernier A. Ricaud K. Bernadet M.-D. Gontier K. Davail S. (2017). Pre- and post-prandial expression of genes involved in lipid metabolism at the end of the overfeeding period of mule ducks. Mol. Cell Biochem. 438 (1–2), 111–121. 10.1007/s11010-017-3118-6 28766168

[B33] Théron L. Astruc T. Bouillier-Oudot M. Molette C. Vénien A. Peyrin F. (2011). The fusion of lipid droplets is involved in fat loss during cooking of duck “foie gras. Meat Sci. [Internet] 89 (4), 377–383. 10.1016/j.meatsci.2011.04.018 21621925

[B34] Trico D. Mengozzi A. Frascerra S. Scozzaro M. T. Mari A. Natali A. (2019). Intestinal glucose absorption is a key determinant of 1-hour postload plasma glucose levels in nondiabetic subjects. J. Clin. Endocrinol. Metab. 104 (6), 2131–2139. 10.1210/jc.2018-02166 30445459

[B35] Wankar A. K. Rindhe S. N. Doijad N. S. (2021). Heat stress in dairy animals and current milk production trends, economics, and future perspectives: The global scenario. Trop. Anim. Health Prod. 53 (1), 70. 10.1007/s11250-020-02541-x 33398462

[B36] Yahav S. McMurtry J. P. (2001). Thermotolerance acquisition in broiler chickens by temperature conditioning early in life - the effect of timing and ambient temperature. Poult. Sci. 80, 1662–1666. 10.1093/ps/80.12.1662 11771878

