# Supplementary material for: Optimization of embryonic thermal programming confirms increased liver fattening in mule ducks and changes in lipid metabolism
Source: Front Physiol. 2023 May 18;14:1142398. doi: 10.3389/fphys.2023.1142398 (PMC10233139; doi:10.3389/fphys.2023.1142398)
Supplement: Supplementary file 1 [file Table1.DOCX]

| **Gene name** | **Gene Symbol** | **main function** | **Primer sequence (F & R)** |
| --- | --- | --- | --- |
|  |  |  |  |
| Acyl-CoA Dehydrogenase Family Member 11 | ACAD11 | Acyl-coA dehydrogenase enzyme | TGGTTGTACCTCGAGCTGTG CATCCACATGAGAGGGCTTT |
| Acetyl-CoA Acetyltransferase 1 | ACAT1 | Acetoacetyl-CoA synthesis | GGTATCCCCGTGGAAGAAGT TTCCACCAGCAACCATTACA |
| Acetyl-CoA Carboxylase | ACC | Carboxylation of acetyl-CoA | TGCCTCCGAGAACCCTAA AAGACCACTGCCACTCCA |
| ATP Citrate Lyase | ACLY | Acetyl-CoA and oxaloacetate synthesis | ACCCCACTGTTGGACTATGC GCTTCAAGCGCTTCTGATCT |
| Acyl-CoA oxidase 1 | ACOX1 | Fatty Acid beta-oxidation | CATGTTTGAGTGGGAAAGA TTTTCAGGGCAGGAAAATTG |
| Acyl-CoA Synthetase Long Chain Family Member 1 | ACSL1 | Lipid synthesis | GGCTGGCTTCATACAGGAGA CTCTTTTCTTGGCCCATTTG |
| Aldehyde Dehydrogenase 7 Member A1 | ALDH7A1 | Oxidation of alcohol molecules | GGAAGCTTGGTCTCATTGGA ATGGCCAGGTCTTTCTGAAG |
| Apolipo protein B | APOB | Transport of lipids | TCTCACCGTGACTTGAGTGC TCCCAGCAGAAGGTGAAGAT |
| Choline/Ethanolamine Phosphotransferase 1 | CEPT1 | Choline/Ethanolamine synthesis | CTGCTGTGCAGCTCTTTGAA CGCAGTATCGAAGCAAATCA |
| Carbohydrate-responsive element-biding protein | ChREBP (MLXIPL) | glucose-induced lipogenesis transcription factor | TCCTCCACACTGCAAAACTG ACCATGCCGTTGAAAGACTC |
| Activating Transcription Factor 2 | CREB2 ATF2 | Regulator of gene transcriptions | CAGGTCCTTTTCCTCTGCTG CCTGGGATTCCTGGGATACT |
| Diacylglycerol O-Acyltransferase 2 | DGAT2 | Triglycerides synthesis | TGGGGCTTGTTACCGTACTC TGGAGAAGATGGGCTGAATC |
| Fatty Acid Elongase 6 | ELOVL6 | Fatty acids elongation | CTACTTTGTGCTCTTCTGCCATTTT TGGCTTCTGTCTCATTTCTACCAC |
| Enolase 1 | ENO1 | Glycolysis | CGCTACATGGGGAAAGGTGT AAGGTCAGCAATGTGACGGT |
| Fatty acid synthase | FAS | Fatty acid synthesis | TGAAGAAGGTCTGGGTGGAG CTCCAATAAGGTGCGGTGAT |
| Glyceraldehyde-3-phosphate Dehydrogenase | GAPDH | Phosphorylation of glyceraldehyde-3-phosphate | CAGAGGACCAGGTTGTCTCC CACCACACGGTTGCTGTATC |
| Glucose transporter 2 | GLUT2 | Transport of glucose | GGAGTTGACCZZCCCGTTTA CCCACCTCGAAGAAGATGAC |
| Glycerol-3-phosphate acyltransferase 1 | GPAT1 | Lipid synthesis | ACAACTTCAGCGGTCCTGTT GCGCTGAGGTAGGAACGTAG |
| Hexokinase 1 | HK1 | Glycolysis | GAGGGAGCAGACGTTGTGAA GCCGCATCTCCTCCATGTAA |
| Hexokinase 2 | HK2 | Glycolysis | GTCCACTGCATCTCCGACTT CACATCCAGGTCGAACTCCT |
| Lipase C, Hepatic Type | LIPC | Triglycerides lipase | TCTGCCCATCACAGTTCTTG TGTTTCCCCTGCTTTCACTC |
| Nuclear Receptor Subfamily 1 Group H Member 3 | LXRA | Macrophage regulator | CATCTCGACGCTACAATCCA GTGTGCTGCAGTCTCTCCAC |
| Malic enzym | ME | Oxidative decarboxylation of malate | GCTTGATAGTGAAGGGGCGT AGGATAGGTGGAGGCAGTGT |
| Pyruvate Dehydrogenase E1 Subunit Alpha 1 | PDHA1 | Pyruvat conversion in Acetyl-CoA | CTGCAGAGCTGGAAAAGGTC CACTGCCACATCAATTTCCTT |
| Perilipin 2 | PLIN2 | Lipid droplets synthesis | CAAACCTTCCTTTGGTGAGC TTGTCCAGACCCATACATGC |
| Peroxisome proliferator activated receptor alpha | PPARA | Beta-oxidation regulator | GGATGCTGGTAGCCTATGGA ACGTGCACAATGCTCTCTTG |
| Peroxisome proliferator-activated nuclear receptor gamma | PPARg | Regulator of lipid storage | CCCAAGTTTGAGTTCGCTGT GCTGTGACGACTCTGGATGA |
| Stearoyl-CoA desaturase 1 | SCD1 | fatty acid biosynthesis (oleate and palmitoleate) | AGTGCTGCTCACATGTTTGG TGAAGTCGATGAAGGCTGTG |
